# Mapping post-Brexit environmental law

**DOI:** 10.1007/s12027-020-00627-5

**Published:** 2020-09-17

**Authors:** Colin T. Reid

**Affiliations:** University of Dundee, Park Place, Dundee, DD1 4HN UK

**Keywords:** Brexit, Environment, Environmental governance, Environmental principles, Devolution

## Abstract

The UK’s withdrawal from the EU will not bring about immediate changes to the substance of environmental law in the UK, but that law will become easier to change. The future position is complicated by devolution within the UK, where differing policy objectives on continuing alignment with the EU and weaknesses in the inter-governmental structures are causing problems. Environmental principles are being given legal recognition and new structures for environmental governance being created for each nation. These include environmental watchdogs that go some of the way to making up for the loss of the oversight provided by the EU institutions.

## Background

When I participated in a conference in 2015, almost a year before the Brexit referendum and when the idea of the UK leaving the EU was a speculative thought experiment, not a practical reality, a number of key issues for environmental law were identified:^1^

- would the legislative slate be wiped clean, with all the law coming from the EU having to be removed and replaced, or would existing EU law be carried over and continue in force?
- would the future see major divergence between EU and UK laws, and in particular would the UK embark on a programme of deregulation?
- would environmental law become more volatile and subject to short-term shifts in policy?
- in the absence of the European Commission (Commission) and the Court of Justice of the European Union (CJEU), how would the government be held to account for its environmental performance and its success or failure in meeting targets?

These questions are considered in the rest of this paper and at the time of writing in August 2020, not all of them have been answered, even though Brexit has now formally taken place. The legal basis for this in the UK is the European Union (Withdrawal) Act 2018 (Withdrawal Act) (passed before there was any Withdrawal Agreement settled between the UK and the EU). This has now been amended by the European Union (Withdrawal Agreement) Act 2020 (2020 Act), reflecting the terms of the Agreement made in late 2019 between the Johnson government and the EU.^2^ At present we are in the transition or implementation period provided for under that Agreement and although the UK formally left the EU on 31 January 2020, very little has changed. The big change will come at the end of this period, on 31 December 2020. At that date the ties are fully severed, unless the negotiations for a new agreement on future trading and other relations reach a successful conclusion before then.

Looking back at political developments over the past few years within the UK, one of the big surprises is that the environment has featured so strongly in the planning for a post-Brexit future, with an acceptance that substantial measures are needed to fill the gaps created as the UK leaves the EU. At the time of the referendum, when deregulation was very much a theme of the Leave campaign, it looked as if the EU-based elements of environmental law might just be torn up with no replacement. Since then there has been an acceptance across the UK that new measures are necessary to guide the development of environmental law and policy and to ensure that public authorities live up to their environmental commitments. The introduction of major structural features to support environmental protection now seems certain, but at a more detailed level, deregulatory themes continue to be significant.^3^

## Start again or carry-over?

During the period of the UK’s membership of the EU, its environmental law has been transformed and EU measures have played the leading role in this transformation. The law on air pollution, waste, water resources, nature conservation, noise and much more is now to be found predominantly in EU Directives and the domestic legislation introduced to give effect to these. It was very rapidly accepted that it was not possible to remove all EU and EU-based measures from the UK’s legal system nor to start again with a new national regime for every area. The whole point of the supra-national nature of the EU is that EU law is not separate from but is deeply embedded in the national legal systems in various ways and extracting the EU elements was just not feasible.

Accordingly, the only practical decision was to say that all existing EU law, both legislation and judicial decisions, continues in effect.^4^ It now becomes a special form of domestic law, known as “retained EU law”, subject to a number of special procedural rules for an extended internal transitional period. As domestic law, it is now subject to amendment by the UK authorities, and in preparation for Brexit there have been hundreds and hundreds of technical amendments under the Withdrawal Act’s wide powers to make the adjustments necessary to give effect to Brexit,^5^ e.g. to cut out the role of EU institutions in the operation of any regulatory systems.

Even at the end of the transition period, decisions of the CJEU prior to Brexit continue to be binding on UK courts, although the Supreme Court can depart from CJEU decisions on the same basis as it can from its own earlier precedent (the same applies to the High Court of Justiciary in Scotland where it is the final appeal court).^6^ The 2020 Act^7^ has added the possibility of other courts being allowed to overturn CJEU decisions,^8^ but this has been heavily criticised;^9^ if other courts could revisit an issue settled by the CJEU, large areas of the law would become uncertain given how much of EU environmental law rests on judicial decisions (e.g. the application of the precautionary principle in protecting Special Areas of Conservation).

## Divergence and deregulation

Environmental policy is a big issue in the current negotiations on the future arrangements between the UK and the EU. Whereas maintaining equivalent levels of protection in this and other areas was a legal commitment under the Agreement made with Mrs May’s government,^10^ the Withdrawal Agreement that has taken effect^11^ has replaced this legal commitment with vaguer statements in the accompanying Political Statement.^12^ As a gross over-simplification, at present the EU, in exchange for a close relationship and open access to markets, is asking the UK to commit to continuing alignment on environmental and other matters to secure a “level playing field”. In contrast, the UK government says that the whole point of Brexit is to have the freedom to make its own rules and has been unwilling to make such a commitment. In reality, the UK may not want to do much different in substance, but the freedom to do so is ideologically important (at present).

Two factors complicate the position. The first is that in some areas, including the environment, there may actually be a desire for moving away from EU standards. This is not only because that fits the political views of some in government, who see environmental measures as an obstacle to economic recovery and development,^13^ but also because it may be a requirement for securing trade agreements with other partners that do not share the EU’s standards. In particular, reaching a deal with the USA may require adjusting laws on issues such as food production to match their standards rather than the different EU ones.

Of more immediate concern have been the implications of devolution. The environment is one of the areas where the devolved administrations have wide responsibilities (the precise division of powers under the arrangements for each nation varies), but since the UK has a devolved system of government, not a federal one, ultimate power still rests with the UK government. This structure was devised at a time when membership of the EU was not seriously challenged and it is now widely accepted that the structures for inter-governmental relations within the UK were not fully developed. This was largely because EU membership provided shared frameworks, constraining how far the nations within the UK could diverge within the areas of devolved powers. There was scope for national innovation, e.g. the restructuring of environmental governance in Wales,^14^ but always within the bounds set by the EU. This meant that there was little need for detailed mechanisms for agreeing a common approach or resolving disputes between the UK’s nations. Even the UK government’s sole responsibility for foreign affairs, with no formal role for the devolved authorities, was less problematic when it was so often exercisable within the confines of EU policy.

The devolved administrations, especially Scotland, argued that any power in devolved areas (which includes most environmental matters) should pass straight from Brussels to Edinburgh, Cardiff and Belfast. However, the Withdrawal Act allows the UK government to keep hold of power in any area where it thinks that a common position across the whole UK is desirable, even though the subject-matter is normally devolved.^15^ The intensity of the political storms over this is shown by the fact that the 2020 Act approving the Withdrawal Agreement and activating these provisions was passed without the consent of any of the devolved Parliaments, something which is not legally necessary but which the devolution legislation says is “normally” expected for measures that have an impact on devolved matters.^16^ Such tensions were reinforced in the summer of 2020 by the UK government’s proposals on an Internal Market,^17^ based on a strong and wide application of the principles of mutual recognition and non-discrimination. This would still allow the devolved authorities to set their own standards, but at the risk having them undermined in practice by the need to accept goods produced to the potentially different standards set for the economically dominant English market. For example, producers in Scotland subjected to new restrictions introduced by the Scottish Government to protect the environment would face being undercut by producers from England, operating free from these restrictions, taking advantage of their right to access the Scottish market.

Alongside the constitutional arguments of how power should be distributed, there are sharp policy differences. The current Scottish Government is committed to “dynamic alignment” with the EU, maintaining existing standards and keeping in step with developments in the EU (with a view to an independent Scotland being able to rejoin the EU).^18^ This is in marked contrast to the UK government’s desire to “take back control” and have a free hand to raise or lower standards as it thinks fit (or as necessary in the light of trade deals with other partners). A further complication arises from the special provisions dealing with the island of Ireland. These mean that Northern Ireland is legally bound to dynamic alignment with the EU by virtue of its unique position under the Withdrawal Agreement, maintaining its links with the EU customs union and single market. There are thus considerable tensions over the future direction of environmental regulation.

At a more basic level, the fact that environmental matters are devolved means that each nation has been making its own responses to the issues arising from Brexit. Although there have been repeated statements about the desirability of co-operation and collaboration, in fact each nation has proceeded on its own timetable, with the UK government able to move faster in producing a detailed scheme for the future. This means that a fragmented picture of environmental governance is inevitable. The UK Government (with responsibility for England and non-devolved matters) has produced detailed proposals that fill the gaps left by withdrawal from the EU. These are included as part of wider environmental legislation going through Parliament during 2020; the Environment Bill.^19^ Northern Ireland (which lacked an operational Assembly and Government between January 2017 and January 2020) is joining these arrangements.^20^ Scotland has developed its own proposals to fill the gaps, and again these are undergoing the legislative process during 2020; the UK Withdrawal from the European Union (Continuity) (Scotland) Bill (Continuity Bill).^21^ In Wales, the future policy is not yet clear and any legislative proposals have been deferred until after the elections in May 2021.^22^

## Volatility

A notable feature of EU law-making processes is that they are slow. This means that they lack flexibility, but for large-scale environmental problems this can be a virtue since once the law is made it is hard to change. This enables fixed and long-term targets to be established to deal with problems such as water quality, climate change and waste reduction, where long-term action involving many parties is needed to improve the position.

Now that the UK will be acting on its own, it will be able to change the law much more quickly. This enables a fast response to changing circumstances, but also leaves the law much more vulnerable to short-term political pressures. For example, in the past a long-term plan for regular increases in the tax on petrol, to combat air pollution and climate change, never came into operation because there was always a temporary reason for not increasing the tax in any particular year.

Some steps are being taken towards longer-term planning. In particular, the current Environment Bill requires the UK government to produce an environmental improvement plan “for significantly improving the natural environment” over a period of at least 15 years.^23^ Progress will have to be reported annually and it is to be accompanied by a number of statutory long-term targets in various sectors, including the priority areas of air quality, water, biodiversity, and resource efficiency and waste reduction. The process of developing these is just beginning,^24^ but they are likely to be less extensive than the objectives set under EU laws such as the Water Framework Directive^25^ (the statutory requirement is for targets on just “at least one matter within each priority area”), as well as being more vulnerable to future adjustment. An Environment Strategy for Scotland is also being developed,^26^ but without the legal basis of the environmental improvement plan and at its present stage it is focused on broad objectives rather than specific legal targets.

The increased volatility arising from the change from EU to UK law-making also brings a constitutional issue for the devolved administrations. Within the EU they had to follow EU law, but that was made by a formal, open and consensual process. Although it was the UK as the Member State that had the formal decision-making role, the early stages of the process were transparent and there were ways in which the devolved administrations could engage in them. Now they will often be bound (formally or in practice as a result of the dominance of the English market) by whatever is decided in London, with no proper inter-governmental procedure to ensure that attention is paid to the different national views. Recent experience on a range of matters has shown that important matters can be decided without proper notice to the devolved administrations, let alone meaningful consultation or agreement.^27^

## Principles

A further feature affecting the future shape of environmental law in the UK is the role and status of environmental principles. The nature of the arrangements for carrying over EU law means that the environmental principles embedded in the EU treaties would be lost if not specifically recognised in some way. Both the UK and the Scottish governments have been willing to give legal recognition to the four key environmental principles – preventive, precautionary, rectification at source and polluter pays – with England further giving express recognition to the principle of integrating environmental concerns into all areas of policy.

The details of the proposals differ. Under the Environment Bill (for England and Northern Ireland), this recognition is taking the form of a legal duty to prepare a policy statement on the principles, with a duty on Ministers to “have due regard” to this in making policy.^28^ In Scotland, the obligation in making policy is to “have regard” to the principles themselves, assisted by ministerial guidance.^29^ Moreover, this duty applies not just to the Scottish Ministers but to all public authorities developing policies covered by the Environmental Assessment (Scotland) Act 2005. That Act applies the process of strategic environmental assessment based on EU law to most policy making, not just to the categories of plans and programmes covered by the EU Directive,^30^ so that the scope of the duty in Scotland will be much wider. Nevertheless, the exclusion of budgetary plans has been noted, especially in the context of arguments for a “green recovery” and “building back better” from the current Covid-19 recession. Critics are arguing for a wider and stronger duty, that all public authorities should be required to apply, or act in accordance with, the principles in everything they do (e.g. taking individual regulatory decisions), not just in policy-making.^31^

## Accountability

In considering the consequences of Brexit on environmental governance, the loss of the Commission’s oversight and ultimately the CJEU’s power to compel compliance was widely recognised as one of the most significant impacts.^32^ This removes important mechanisms for holding the government to account, not least because of the high cost of legal actions in the UK. The need for something to fill this gap has been accepted, but different plans have been developed in the different parts of UK at different speeds. Such variation is in part necessary to fit with the separate governance and court structures in each nation, but the UK government’s speed in making its own plans left little opportunity for discussion of an integrated approach with the other administrations. The emerging picture, therefore is of a patchwork of different arrangements, and there is a strong case for a more considered review of how well these are working together once the dust has settled.^33^ Furthermore, it seems impossible for the new arrangements to be fully operational on a legislative basis by the time Brexit takes full effect at the end of 2020 and non-statutory interim arrangements are likely.

The Environment Bill before the UK Parliament provides for the creation of the Office for Environmental Protection (OEP), an independent body to review government policy and receive and investigate complaints of non-compliance with environmental law.^34^ This will cover things done by the UK government for the whole of the UK and for England. Northern Ireland has agreed that the Office for Environmental Protection will also cover devolved matters there. Scotland is proposing its own oversight body, Environmental Standards Scotland (ESS).^35^ Wales has a different governance framework for environmental matters, based on the Well-Being of Future Generations (Wales) Act 2015, and detailed plans for the future are being left until after the May 2021 elections.^36^

No domestic body can match the independence of the powerful external bodies provided by the EU, but there should be structures to provide for the oversight arrangements as strong a guarantee of independence from government as is possible. The fact that appointments are in the hands of ministers for the OEP has been criticised and although in Scotland appointments will have to be approved by the Scottish Parliament, the ministers still have some role. In both cases governmental control over the resources provided to the oversight bodies has also raised concerns. Moreover, the OEP is going to have multiple roles, being involved in reviewing progress on environmental plans and advising government, as well as handling complaints, creating a possible conflict of interests in the oversight body.

In terms of the accountability mechanisms, there are significant differences between what is proposed for the OEP and ESS. The OEP will have a role in monitoring progress in improving the environment in line with the environmental improvement plan as well as progress towards the statutory targets set. In this capacity it will produce annual progress reports. It will also have the power to report on any matter relating to the implementation of environmental law; these reports are to be laid before Parliament and require a ministerial response. The OEP will also have a role in relation to failures to comply with environmental law. It can act if it considers that there is a serious failure by a public authority to comply with the law, issuing a decision notice identifying the failure and specifying the steps to be taken to remedy the breach. The authority receiving it must produce a written response to the notice, but the notice itself is not directly enforceable. The OEP can, however, seek an environmental review before the Upper Tribunal of the authority’s original (non-)action which amounts to the failure to comply with environmental law. This is to proceed on the basis of the standard judicial review principles and remedies, thereby limiting review to issues of legality, not merits, and excluding the possibility of fines or other sanctions as a result of the non-compliance. This structure has been criticised as falling well short of the substantial powers wielded by the Commission and CJEU.

For ESS, acting on its own initiative or on receipt of complaints, its role will be to monitor the effectiveness of environmental law and its implementation as well as public authorities’ compliance with environmental law. Its monitoring extends to considering the implementation of international obligations (which under the UK’s dualist approach create legal rights and duties only if and when incorporated by parliamentary legislation). ESS will have the power to issue an improvement report where it finds a failure to comply with, or to apply or implement effectively, environmental law. In response to such a report the Scottish Ministers must prepare an improvement plan setting out the measures to be taken, the timescale and monitoring arrangements. This plan must be presented to Parliament and can be rejected, requiring a revised plan to be produced. In the event of non-compliance with the law that is causing environmental harm or a risk of such harm, ESS will be able to issue a compliance notice requiring the authority concerned to take remedial steps. The authority can appeal to the sheriff court against a compliance notice. In the event of the authority failing to comply with a notice without reasonable excuse, the matter can be referred to the Court of Session which can order further enforcement measures and treat the failure as a contempt of court, opening the path to substantial penalties. In serious cases, ESS can also seek judicial review.

This amounts to a considerably wider and stronger system of oversight than for the OEP, but a number of concerns have been raised. The definition of “environmental law” is potentially rather narrow, both in itself (some environmentally significant matters may not involve provisions “mainly concerned with environmental protection”) and because the Bill excludes climate targets and access to environmental information. In both of these areas there are other monitoring and reporting structures, but their exclusion prevents a holistic view of environmental matters being taken. Similarly, ESS is not to act on the basis of failures arising from an individual regulatory decision. This is designed to prevent overlap with existing appeal and review mechanisms and to focus attention on strategic and widespread concerns, but the experience with the Commission and CJEU is that it can be an individual case, not a systemic issue, that raises significant points requiring attention.

With the legislation for these new bodies still being debated, and proposals in Wales still to emerge, it is too early to make definitive statements about how effectively the new structures will fill the governance gap left by the loss of the EU procedures. For all that there are criticisms of aspects of these, one really positive point must be emphasised. Around the time of the referendum, when deregulatory rhetoric held sway, it seemed unlikely that any mechanisms would be put in place to replace the oversight exercised by the EU institutions. The current proposals may not be perfect but do go a long way to ensuring that the governments will still be held accountable for their environmental performance.

## The future

Although the UK has now formally left the EU, the future still remains deeply uncertain, even more so since Covid-19 has disrupted all political processes. A lot depends on the outcome of the negotiations on the future relationship between the EU and the UK and what priorities the UK has in relation to other international relations and trade agreements. Until the position on those is clearer, there will remain uncertainty over the future direction of the UK’s environmental policy and the extent of alignment with the EU on environmental (and other) standards and policies. There is also uncertainty over the effectiveness of the new oversight mechanisms currently being established.

Further problems are arising because of devolution. The constitutional arguments over where power should lie are exacerbated by the fact that the Scottish and UK governments have different ambitions for the future relations with the EU and the resulting tensions are placing great strain on the structures for inter-governmental relations. These structures were created on the basis of continuing EU membership, and therefore designed to deal with only limited divergence between the different administrations, all operating within the same EU “envelope”. They were not designed to cope with the significantly different plans and policies being pursued as at present. For Wales, detailed environmental governance proposals are still to emerge, whilst the full impact of Northern Ireland’s unique position, committed to close ties with the EU, still remains to be worked out, in practical and policy terms.

As has been the case for the past four years, there are lots of moving parts to consider. Few of them are coming a halt and even when they do it is not in an order that allows for new frameworks for environmental policy and governance to be logically assembled. As has been the case for too long now, the only certainty is more uncertainty.

## Footnotes

1 See *Reid* [1], [2].

2 Agreement on the withdrawal of the United Kingdom of Great Britain and Northern Ireland from the European Union and the European Atomic Energy Community (2019/C 384 I/01).

3 *Branson* [3].

4 European Union (Withdrawal) Act 2018, ss 2-7.

5 Ibid., s 8.

6 Ibid., s 6.

7 European Union (Withdrawal Agreement) Act 2020, s 26, amending s 6 of the Withdrawal Act.

8 *Ministry of Justice* [4].

9 For example, *Reid* [5].

10 Draft Agreement on the withdrawal of the United Kingdom of Great Britain and Northern Ireland from the European Union and the European Atomic Energy Community (2018) https://ec.europa.eu/commission/publications/draft-agreement-withdrawal-united-kingdom-great-britain-and-northern-ireland-european-union-and-european-atomic-energy-community-agreed-negotiators-level-14-november-2018_en.

11 Note 2 above.

12 Political declaration setting out the framework for the future relationship between the European Union and the United Kingdom (2019/C 384 I/02 178).

13 Note 3 above.

14 Well-being of Future Generations (Wales) Act 2015.

15 European Union (Withdrawal) Act 2018, s 12.

16 E.g. Scotland Act 1998, s 28(8).

17 *Department for Business, Enterprise and Industrial Strategy* [6].

18 Policy Memorandum on the UK Withdrawal from the European Union (Continuity) (Scotland) Bill (SP Bill 77), paras 20-33.

19 Environment Bill (HC Bill 9 of 2019-21); detailed references are to the Bill as first introduced.

20 Environment Bill, Scheds 2 and 3.

21 UK Withdrawal from the European Union (Continuity) (Scotland) Bill (SP Bill 77); detailed references are to the Bill as first introduced.

22 First Minister’s Statement on the Legislative programme; Record of Proceedings of Senedd, 15.7.2020; Plenary para.334.

23 Environment Bill, cll 1-14.

24 DEFRA [7].

25 Directive 2000/60/EC of the European Parliament and of the Council of 23 October 2000 establishing a framework for Community action in the field of water policy [2000] OJ L 327/1.

26 Scottish Government [8].

27 For example, opening statement by Cabinet Secretary Roseanna Cunningham to the Environment, Climate Change and Land Reform Committee of the Scottish Parliament on 29 October 2019 http://www.parliament.scot/parliamentarybusiness/report.aspx?r=12328&i=111406&c=2225301&s=.

28 Environment Bill, cll 16-18.

29 Continuity Bill, ss 9-14.

30 Directive 2001/42/EC of the European Parliament and of the Council of 27 June 2001 on the assessment of the effects of certain plans and programmes on the environment [2001] OJ L 197/30.

31 *House of Commons Environment, Food and Rural Affairs Committee* [9], paras 27-43.

32 *UKELA* [10], *Round-Table* [11].

33 *House of Commons Environment, Food and Rural Affairs Committee* [9], para.139.

34 Environment Bill, cll 21-40.

35 Continuity Bill, ss15-41.

36 Note 22 above.
